# Skin Microbiomes of California Terrestrial Salamanders Are Influenced by Habitat More Than Host Phylogeny

**DOI:** 10.3389/fmicb.2018.00442

**Published:** 2018-03-14

**Authors:** Alicia K. Bird, Sofia R. Prado-Irwin, Vance T. Vredenburg, Andrew G. Zink

**Affiliations:** ^1^Department of Biology, San Francisco State University, San Francisco, CA, United States; ^2^Department of Evolution and Ecology, University of California, Davis, Davis, CA, United States; ^3^Department of Organismic and Evolutionary Biology, Harvard University, Cambridge, MA, United States

**Keywords:** microbiome, amphibian, symbiosis, bacteria, *Ensatina eschscholtzii*, *Batrachoseps*

## Abstract

A multitude of microorganisms live on and within plant and animal hosts, yet the ecology and evolution of these microbial communities remains poorly understood in many taxa. This study examined the extent to which environmental factors and host taxonomic identity explain microbiome variation within two salamander genera, *Ensatina* and *Batrachoseps*, in the family Plethodontidae. In particular, we assessed whether microbiome differentiation paralleled host genetic distance at three levels of taxonomy: genus and high and low clade levels within *Ensatina eschscholtzii*. We predicted that more genetically related host populations would have more similar microbiomes than more distantly related host populations. We found that salamander microbiomes possess bacterial species that are most likely acquired from their surrounding soil environment, but the relative representation of those bacterial species is significantly different on the skin of salamanders compared to soil. We found differences in skin microbiome alpha diversity among *Ensatina* higher and lower clade groups, as well as differences between *Ensatina* and *Batrachoseps*. We also found that relative microbiome composition (beta diversity) did vary between *Ensatina* lower clades, but differences were driven by only a few clades and not correlated to clade genetic distances. We conclude this difference was likely a result of *Ensatina* lower clades being associated with geographic location and habitat type, as salamander identity at higher taxonomic levels (genus and *Ensatina* higher clades) was a weak predictor of microbiome composition. These results lead us to conclude that environmental factors are likely playing a more significant role in salamander cutaneous microbiome assemblages than host-specific traits.

## Introduction

Just as plants and animals have evolved and adapted to particular habitats, forming complex interconnected communities, microbial species have also evolved to form communities that are particularly well-suited to specific environments. These unique microbial communities (here on referred to as microbiomes) can be found in a range of environments from hot springs (Jackson et al., 2001) to vertebrate digestive tracts (Ley et al., 2008a,b). These microbiomes can play a crucial role in the health and well-being of their host (Moloney et al., 2014). Microbiomes contribute to host immunity, physiology, development, and behavior (McFall-Ngai et al., 2013). Despite a growing understanding of the function and complexity of microbiomes, we are just beginning to understand the factors that cause microbiomes to vary across host individuals, populations, and closely related species. Technological advancements in DNA sequencing have made it possible to get a more complete picture of microbiome community composition (Petrosino et al., 2009), and begin answering questions about variation in the host–microbiome relationship (Spor et al., 2011; Council et al., 2016; Moeller et al., 2016).

The amphibian skin microbiome has been a focus of recent research, due to the non-invasive method of sample collection as well as the relevance of skin microbiomes to amphibian health (McKenzie et al., 2012; Jani and Briggs, 2014; Kueneman et al., 2014). The skin of an amphibian is essential for the proper function of many biological processes, including moisture balance, gas exchange, and disease defense. Proper function of skin-related biological processes depends on host factors (physiology, metabolism, behavior, etc.) as well as the skin microbiome, which often provides essential benefits that affect host fitness. For example, in defense against the fungal pathogen, *Batrachochytrium dendrobatidis*, some amphibian species release antimicrobial peptides from glands that can inhibit *Batrachochytrium dendrobatidis* growth (Rollins-Smith and Conlon, 2005), while other hosts might also, or alternatively, harbor beneficial microbes, such as *Janthinobacterium lividum*, on their skin that protect the host from infection (Harris et al., 2009).

Despite evidence that microbiomes are often essential components of host fitness, the relative influence of environment versus host taxonomy in shaping amphibian microbiomes remains unclear. In aquatic amphibians, for example, evidence suggests host taxonomic identity more strongly predicts microbiome composition than environment (McKenzie et al., 2012; Walke et al., 2014; Kueneman et al., 2014). This result indicates there may be an evolutionary relationship between hosts and their microbiomes. For example certain taxonomic groups may harbor specific microbes that are passed vertically or horizontally among closely related hosts due to their unique life histories (Walke et al., 2014). Alternatively, unique taxonomic groups may have a genetic predisposition to acquire a biased subset of microbes from the environment. In terrestrial salamanders, habitat (rather than host taxonomy) seems to be the strongest predictor of microbiome composition (Fitzpatrick and Allison, 2014; Loudon et al., 2014; Muletz Wolz et al., 2017; Prado-Irwin et al., 2017), lending evidence to the theory that some amphibians may simply acquire skin associated microbes directly from their surrounding environment. Differences in the relative influence of host phylogeny is likely due to biological differences in aquatic versus terrestrial amphibians, but may also be due to a lack of studies with broad habitat sampling of terrestrial salamanders. However, one study on plethodontid salamanders did find good evidence that site rather than host species identity explained skin microbiome beta diversity patterns (Muletz Wolz et al., 2017). Further studies, such as the one we present here, focusing on groups with well-characterized phylogenies and geographic ranges encompassing several habitat types can help elucidate which factors influence the amphibian skin microbiome.

The terrestrial salamander *Ensatina eschscholtzii* (family Plethodontidae) is an ideal study system for exploring the relative influences of host taxonomy and environment on the amphibian skin microbiome. The evolutionary history of *Ensatina* as a ring species has been well established (Stebbins, 1949; Wake and Yanev, 1986; Moritz et al., 1992; Kuchta et al., 2009b): the species originated in Northern California and Southern Oregon and later dispersed (divergently) down both the coastal and inland regions of California, eventually meeting again in southern California (Stebbins, 1949; Moritz et al., 1992). Throughout its range *Ensatina* differentiates into 12 distinct genetic clades encompassing seven taxonomic subspecies (Kuchta et al., 2009a). This well-characterized genetic history provides a unique opportunity to assess the degree of similarity of microbiomes among clade groups with varying degrees of genetic distance. In addition, for much of its range, *Ensatina* also overlaps with other plethodontid species within the genus *Batrachoseps*, allowing for a within-family comparison. *Ensatina* and *Batrachoseps* co-occur across several different habitat types, providing an opportunity to disentangle host taxonomic versus environmental influences on the microbiome. If host taxonomy is a primary driving factor in determining the microbiome, we would expect that sympatric species would still harbor distinct microbiome communities. However, if environment plays a more important role, we would expect sympatric species to exhibit more similar microbiomes that more closely related allopatric individuals.

With *Ensatina* and *Batrachoseps* as our study system, we used 16S amplicon sequencing to evaluate to relative roles of host identity and habitat on the skin bacterial community (from here on referred to as the microbiome) of California terrestrial salamanders. The primary objective of our study was to assess whether varying degrees of host genetic distance could explain differences in the skin microbiome. We hypothesized that the microbiome would track the phylogeny of their hosts, with more closely related salamander groups harboring more similar microbiome communities. Correspondingly, we expected more distantly related salamander groups to exhibit more distinct microbiome communities from one another. To test this hypothesis, we assessed differences in the microbiome within the plethodontid family using different genera (*Ensatina* vs. *Batrachoseps*) and species (*Ensatina* and three species of *Batrachoseps*). We also looked at differences in the microbiome within the species *Ensatina* at two clade levels (referred to here as “higher clades” and “lower clades”) as identified in Kuchta et al. (2009a). In addition to determining whether host phylogeny influenced skin microbiomes, we also considered the role of habitat in influencing microbiome variation. We predicted that habitat would explain some variation, but to a lesser degree than host taxonomic identity, as seen in previous studies (Kueneman et al., 2014). Lastly, we compared salamander microbiomes to their surrounding soil microbiomes and hypothesized that the soil microbiome would house a greater diversity of microbial species, as soil and sediment have been shown to have enormous microbial diversity (Torsvik et al., 2002). We also expected the salamander skin microbiome to share some but not all microbial species with the soil, while also housing some unique microbial species not found in the soil as seen in previous work (Prado-Irwin et al., 2017). This would lend evidence to the theory that some degree of vertical or horizontal transmission may be occurring in these species.

## Materials and Methods

### Field Sampling

**FIGURE 1:**
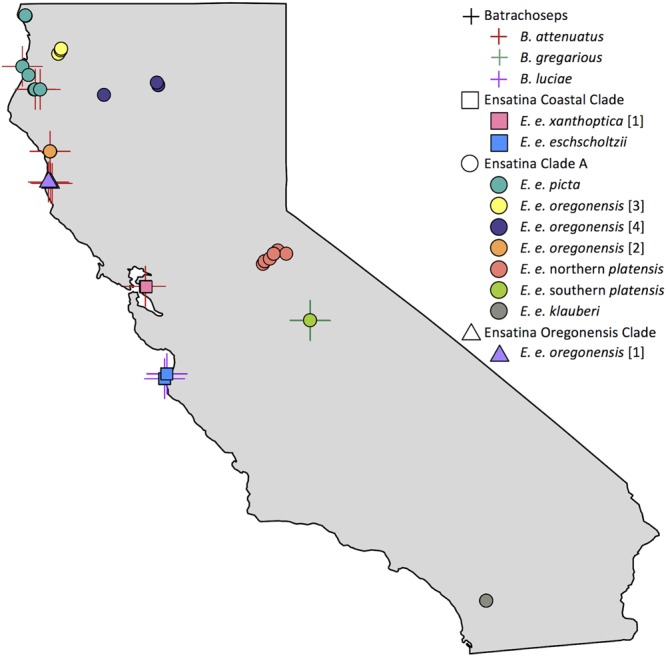
Map of *Batrachoseps* and *Ensatina* sampling localities within California. *Batrachoseps* samples represented with a cross symbol, species distinguished by color. *Ensatina* higher clade groups differentiated by shape (Coastal Clade, square; Clade A, circle; Oregonensis Clade, triangle), lower clade groups distinguished by color. Some symbols overlap due to close proximity of samples. Note that samples from lower clade groups *E. e. croceater* and *E. e. xanthoptica* [2] were not collected in this study. This figure created in R (Becker and Wilks, 2016; R Core Team, 2016).

We sampled 118 salamanders during the rainy season from March – May 2014 and December – February 2015 (**Figure 1** and Supplementary Table 1). Skin microbiome samples were collected from 10 of the 12 distinct genetic clades (here on referred to as “lower clades”) of *Ensatina eschscholtzii* (Kuchta et al., 2009a) (**Figure 1**). These lower clades are nested within three higher clades [Coastal Clade (*n* = 22), Oregonensis [1] (*n* = 10), and Clade A (*n* = 54)], which was an additional level of genetic relatedness between hosts that we compared microbiomes across (Kuchta et al., 2009a). The molecular phylogeny and corresponding range map of these higher and lower genetically distinct clade groups can be found in Kuchta et al. (2009a). Clade identity was assumed based on morphology and known localities, and populations sampled for each clade were sufficiently geographically distant from populations of other clades to avoid hybrid individuals (Kuchta et al., 2009a). The lower clade *E. e. croceater* was not sampled because no individuals were found, likely due to extreme drought conditions within their range during the time of this study. The lower clade *E. e. xanthoptica* [2] was also not sampled because its range includes a high degree of geographic overlap with other clades, and we wanted to avoid sampling hybrid individuals. We also collected skin microbiome samples from *Batrachoseps* where they were found sympatrically with *Ensatina*. Sample size for each *Ensatina* lower clade and *Batrachoseps* species are summarized by location in **Table 1**.

**Table 1:** Sample sizes (*n*) for each *Batrachoseps* species and *Ensatina* subspecies by site.

Site	Species/subspecies	*n*
Alameda	*Ensatina eschscholtzii xanthoptica* [1]	12
	*Batrachoseps attenuates*	5
Calaveras	*Ensatina eschscholtzii* northern *platensis*	10
Humboldt	*Ensatina eschscholtzii picta*	10
	*Batrachoseps attenuates*	5
Jackson State Forest	*Ensatina eschscholtzii oregonensis* [1]	10
	*Batrachoseps attenuates*	5
Leggett	*Ensatina eschscholtzii oregonensis* [2]	10
	*Batrachoseps attenuates*	5
Monterey	*Ensatina eschscholtzii eschscholtzii*	10
	*Batrachoseps luciae*	8
Palomar	*Ensatina eschscholtzii klauberi*	9
Shasta	*Ensatina eschscholtzii oregonensis* [4]	4
Sierra National Forest	*Ensatina eschscholtzii* southern *platensis*	2
	*Batrachoseps gregarious*	4
Siskiyou	*Ensatina eschscholtzii oregonensis* [3]	9

Due to the difficulty of finding individuals in many of the clades, we could not standardize the total sampling area across clades (i.e., some individuals were found at closer distances to one another than others). For each lower clade group, we attempted to sample ten adult *Ensatina*, a comparable sample size relative to other amphibian microbiome studies (McKenzie et al., 2012; Bataille et al., 2016; Hughey et al., 2017; Walke et al., 2017). If 10 adults could not be found after extensive searching, we collected and included juveniles in our analysis for that *Ensatina* lower clade wherever possible. To ensure that including juvenile *Ensatina* in our analyses did not affect our results, we tested whether life stage explained variation in weighted and unweighted microbiome beta diversity within *Ensatina* and found that it did not explain significant differences (adonis *R*^2^= 0.03, *p* > 0.05). This is consistent with previous findings (Prado-Irwin et al., 2017). All of the *Batrachoseps* sampled were adults.

Individuals were located by turning over cover objects (logs, rocks, etc.). Salamanders were handled with new nitrile gloves and rinsed with approximately 50 mL (for *Ensatina*) or 25 mL (for *Batrachoseps*) of 18 MΩ/cm MilliQ water from a sterile syringe to remove any dirt and non-skin associated (i.e., transient) microbes. The salamander was then swabbed 30 times (10 times on dorsal surface, 10 times on ventral surface, and 5 times on each side) using a sterile fine tip rayon dryswab. The swab was then placed in a sterile Eppendorf tube that was immediately placed on dry ice for temporary storage until the sample could be transferred to a -80°C freezer. Every salamander was then swabbed a second time using the same method to test for the presence of the common amphibian fungal pathogen, *Batrachochytrium dendrobatidis*. We took *Batrachochytrium dendrobatidis* swabs to identify diseased individuals for exclusion from analyses, as *Batrachochytrium dendrobatidis* has been shown to cause significant shifts in the microbiome (Jani and Briggs, 2014; Walke et al., 2015; Bataille et al., 2016). We performed a standard real-time quantitative PCR assay to determine possible presence of fungal infection from *Batrachochytrium dendrobatidis* swabs (Boyle et al., 2004). We ultimately did not need to exclude any samples from analyses because all samples were negative for *Batrachochytrium dendrobatidis*.

For each salamander, we took GPS coordinates at the site of sample collection (Supplementary Table 1). GPS coordinates were later input into the Conservation Biology Institute’s Data Basin platform^1^ to determine habitat type (Scrub Oak Chaparral, Upland Redwood Forest, etc.) for each sample, using land-cover data provided by the “California landcover based on California Natural Diversity Data Base (CNDDB) system” layer (Supplementary Table 1).

For each *Ensatina* individual sampled, we also collected a soil sample in a sterile 2 mL Eppendorf tube from under the cover object where the salamander was found. The soil sample was immediately placed on dry ice for temporary storage until the sample could be transferred to a -80°C freezer. We randomly chose five soil samples per *Ensatina* lower clade to analyze for this study. For lower clade groups where less than five *Ensatina* individuals were found, we included all collected soil samples for that clade in our analyses (one associated with each cover object for each individual).

The protocol for the use of salamanders in this research was approved by the California Department of Fish and Wildlife (SC-12919) and the San Francisco State University Institutional Animal Care and Use Committee (Protocol #A12-07).

### Microbiome DNA Extraction and Sample Processing

Bacterial DNA was extracted from each microbiome swab and soil sample using a PowerSoil Isolation Kit (MoBio Laboratories, Carlsbad, CA, United States). Each swab or soil sample (0.25 g) was placed in a bead tube provided by the kit, and extraction was completed using the manufacturer’s protocol. The V3-V4 region of the bacterial 16S rRNA gene was amplified using Illumina primers (Supplementary Table 2) for each sample using a modified version of the Illumina protocol (we used 30 PCR cycles during the amplification step; Illumina Inc., San Diego, CA, United States). Each extract was amplified in triplicate, resulting in a total volume of 75 μl of amplified product per sample. After amplification, product was cleaned up using Agencourt AMPure XP beads to remove non-target DNA. Cleaned product was then re-amplified according to Illumina protocol using sample-specific Illumina Nextera Index primers (Supplementary Table 2). Indexed product was cleaned up with the same methods used for the amplicon product. Clean indexed product was then run on a gel to confirm the presence of product and to ensure no contamination had occurred. Samples were then quantified using qPCR. Each qPCR consisted of 6 μl KAPA SYBR FAST qPCR Master Mix and 4 μl sample. Samples were then pooled in equimolar concentrations and the pool was quantified using qPCR to confirm concentration, and further diluted if required. The pool was then sequenced at the Department of Biology’s Genomics/Transcriptomics Analysis Core facility at San Francisco State University, on an Illumina MiSeq, using a v2 kit.

### Sequence Analyses

Sequence analyses were conducted using QIIME v1.9.0 (Caporaso et al., 2010b). Default protocol was used unless otherwise indicated. Forward and reverse reads were joined and sequences were filtered using a quality score of Q20 (removes reads with <99% base call accuracy), resulting in approximately eight million sequences. Sequences were then clustered into operational taxonomic units (OTUs) at 97% similarity. We used the open-reference subsampling protocol in QIIME to assign OTU taxonomy using the Greengenes 13_8 reference database^2^ (DeSantis et al., 2006; McDonald et al., 2012). Sequences were aligned using PyNAST (Caporaso et al., 2010a). Aligned sequences have been archived under BioProject accession number PRJNA434592. Any sequences that did not match the reference database were clustered into *de novo* OTUs using UCLUST (Edgar, 2010) and taxonomy was assigned using the RDP Classifier 2.2 (Wang et al., 2007). The final OTU table was then additionally filtered across all samples before analysis to remove rare OTUs with fewer than 100 reads and those represented in only one sample (soil or salamander), which removed OTUs representing less than ∼0.001% of all sequences (Bokulich et al., 2013; Kueneman et al., 2014; Walke et al., 2014; Longo et al., 2015), resulting in a total of 6,576 OTUs. Samples were rarefied by analysis to the number of sequences present in the sample with the lowest number of sequences (see below for rarefaction levels associated with each of the individual analyses).

### Statistical Analyses

We assessed differences in the microbiome across five sampling categories, representing different levels of genetic relatedness: sample type (soil or salamander), genus, species, higher clade and lower clade. Higher clade groups and lower clade groups are identified from previous phylogenetic analyses of *Ensatina* (Kuchta et al., 2009a). Rarefaction levels of sequences per sample by analysis: soil vs. salamander = 7968, *Ensatina* vs. *Batrachoseps* = 9466, *Ensatina* vs. *Batrachoseps* (within overlapping range) = 15514, within *Ensatina* higher and lower clade comparisons = 9466. Refer to Supplementary Table 3 for rarefaction levels used for *Ensatina* pairwise comparisons.

Microbiome alpha diversity metrics calculated in QIIME for each sample included: OTU richness, phylogenetic diversity, Simpson diversity index, Shannon diversity index, and Shannon’s equitability (evenness). We determined normality of alpha diversity data using Shapiro–Wilk tests. We compared alpha diversity between soil and salamanders and *Batrachoseps* and *Ensatina* using two-sample *t*-tests (parametric or non-parametric depending on normality). We used a one-way ANOVA to test for differences in Shannon’s diversity and Kruskal–Wallis tests for differences in the other four diversity metrics across *Ensatina* higher and lower clade groups. All alpha diversity comparisons were done using R (R Core Team, 2016). For each of the five sampling categories defined above we identified dominant OTUs, which were defined as OTUs representing 3% or greater of the total sequences found within that respective sampling category. We also identified the number of OTUs that were unique to each sample type within each of the five sampling categories.

We used unweighted and weighted UniFrac distance metrics to calculate beta diversity in QIIME (Lozupone and Knight, 2005). Unweighted UniFrac distances account for the presence or absence of OTUs within each sample. Weighted UniFrac distances account for the presence or absence of OTUs, as well as relative OTU abundances within each sample. It is important to assess results from both UniFrac metrics (weighted and unweighted) as they each give distinct information about differences between microbial communities (Lozupone et al., 2007). The contribution of sample type, host taxonomy, habitat type, and site to beta diversity was analyzed using adonis in QIIME and plotted using a principle coordinates analysis (PCoA) in R (R Core Team, 2016). For a subset of *Ensatina* lower clade groups, we also tested for correlations between host genetic distance and Unifrac distances using a Pearson’s correlation. We used previously published genetic distances between *Ensatina* populations for this analysis (Wake, 1997; Kuchta et al., 2009b).

For *Ensatina*, we also identified a core microbiome for each lower clade group, defined as all the OTUs found on 90% of samples within that group. We then compared relative core abundances across clades using a Kruskal–Wallis test. We also identified the OTUs that were found on 90% of *Batrachoseps attenuatus* samples. However, we did not assess a core microbiome for *B. luciae* or *B. gregarius* due to their low sample sizes.

## Results

### Differences Between Soil and Salamanders

**FIGURE 2:**
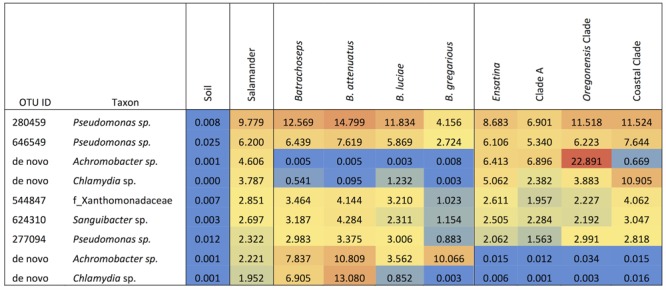
Heat map of the relative abundances as percent values of dominant OTUs (≥3% of microbiome composition) for each sample type, genus, species and *Ensatina* higher clade groups.

Among the 118 salamander samples and the 41 soil samples we analyzed, we found a large degree of variation in OTU richness among individual samples within each of the sample categories. For soil samples, OTU richness varied between 404 and 2072 OTUs per sample (mean = 776 OTUs per sample). For salamanders, OTU richness varied between 201 and 3087 OTUs per sample (mean = 622 OTUs per sample). Soil had significantly greater alpha diversity for all metrics when compared to salamander samples as a whole (non-parametric two-sample *t*-test: OTU richness, *p* = 0.017; phylogenetic diversity, *p* = 0.009; Shannon diversity index, *p* = 0.001; Simpson diversity index, *p* = 0.001). Soil samples were also significantly more even in relative OTU abundances than salamander samples (non-parametric two-sample *t*-test: Shannon’s equitability, *p* = 0.001). Soil samples had no dominant OTUs that made up at least 3% the microbiome across all soil samples. However, 15% of total microbiome across all soil samples was made up of bacteria from three groups: the class Phycisphaerae, and the families Acidobacteriaceae, and Sphingobacteriaceae. By contrast, salamanders (as a whole) had four dominant OTUs, two belonging to the genus *Pseudomonas*, and one to the genera *Achromobacter*, and *Chlamydia*, which collectively made up 24.3% of the total salamander skin microbiome community (**Figure 2**).

The majority of OTUs were found in at least one soil sample and one salamander sample. However, when looking across all soil samples, we did find 94 unique OTUs that were not found in salamander samples. All of these unique soil OTUs were present in very low abundances, all together accounting for approximately 1.1% of the soil microbial community. Conversely, we found 756 OTUs that were unique to salamanders, and not found in any of our soil samples. These unique salamander OTUs were also present in very low abundances, together making up only 3.9% of the total salamander skin microbiome community. Note that while these unique OTU’s might represent bacteria that are acquired from sources other than soil (including conspecifics), it is also possible that they are present in soil that we did not sample at our sites, given that soil is typically quite heterogeneous.

**FIGURE 3:**
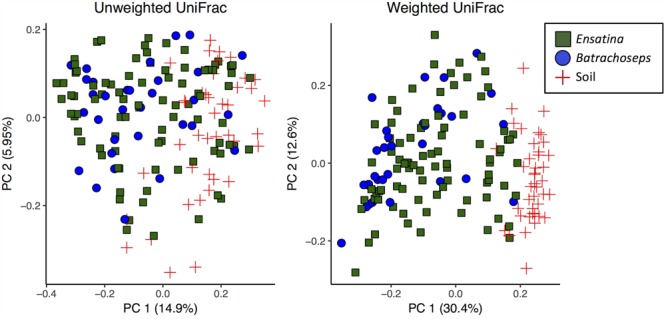
Principle coordinates analysis plots of unweighted and weighted UniFrac distances of all salamander and soil samples.

We assessed beta diversity across all samples (soil and salamander) using unweighted and weighted UniFrac metrics. Analysis of unweighted UniFrac distances across all samples showed that site was the greatest predictor of differences in bacterial communities among samples (adonis *R*^2^= 0.14, *p* = 0.001), with habitat type also explaining a significant amount of variation (adonis *R*^2^= 0.12, *p* = 0.001). Sample type (“Soil” versus “Salamander”) explained a very small degree of variation between samples in an unweighted comparison (adonis *R*^2^= 0.058, *p* = 0.001; **Figure 3**). Conversely, when analyzing the weighted UniFrac distances, sample type (“Soil” versus “Salamander”) was the greatest predictor of differentiation between bacterial communities (adonis *R*^2^= 0.19, *p* = 0.001; **Figure 3**). Site and habitat type also explained a significant amount of variation using the weighted comparison (adonis site *R*^2^= 0.15, *p* = 0.001, habitat type *R*^2^= 0.128, *p* = 0.001) across all samples.

### Differences Within *Ensatina* Higher and Lower Clade Groups

Within *Ensatina* samples, Shannon’s diversity was normally distributed (Shapiro–Wilk test, *p* > 0.05), and all other diversity metrics were non-normally distributed (Shapiro–Wilk test, *p* < 0.001). Simpson’s diversity, Shannon’s diversity and evenness did not differ between lower clade groups (all *p*-values > 0.05). Lower clade groups did differ in OTU richness (Kruskal–Wallis test, *p* = 0.003) and phylogenetic diversity (Kruskal–Wallis test, *p* = 0.001). *Post hoc* pairwise two-sample *t*-tests of lower clade groups showed no significant differences in any alpha diversity metric after Bonferroni correction. No alpha diversity metrics significantly differed between higher clade groups.

**FIGURE 4:**
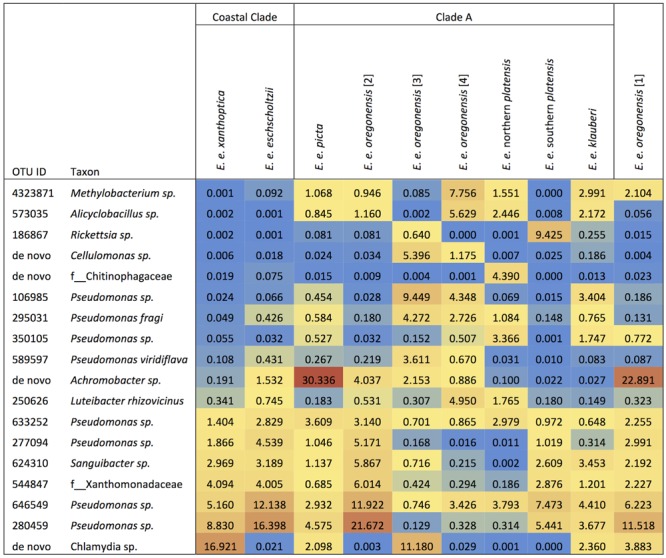
Heat map of the relative abundances as percent values of dominant OTUs (>3% of microbiome composition within sample group) for each *Ensatina* clade.

*Ensatina* lower clade groups harbored between three and seven dominant OTUs (≥3% of microbiome composition), comprising 11.5–57.8% of the microbiome for a given clade (**Figure 4**). Every lower clade group had at least one dominant OTU belonging to the genus *Pseudomonas*. Southern *platensis, oregonensis* [3] and *oregonensis* [4] all had dominant OTUs (OTUs making up >3% of the microbiome) that were not dominant in any other lower clade group. For the higher clade groups, there was a great amount of overlap in dominant OTUs (**Figure 2**). Clade A had three dominant OTUs and the Coastal Clade and Oregonensis [1] Clade each had five dominant OTUs. All higher clades shared two dominant OTUs belonging the genus *Pseudomonas*. Each lower clade group had unique OTUs not present in any other lower clade (Supplementary Table 4). The number of unique OTUs varied between four, as seen in *oregonensis* [4], and 151, as seen in *xanthoptica* [1]. Looking at *Ensatina* samples at the higher clade level, Clade A had 778 unique OTUs, the Coastal Clade had 278 unique OTUs, and *oregonensis* [1] had 26 unique OTUs.

**FIGURE 5:**
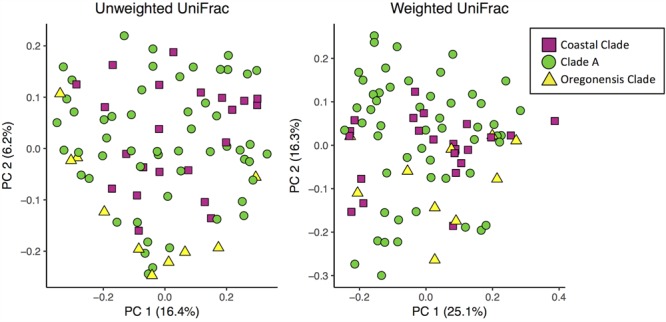
Principle coordinates analysis plots of unweighted and weighted Unifrac distances of *Ensatina* samples by higher clade groups.

Using unweighted UniFrac analyses comparing all *Ensatina* samples, we found that lower clade group and habitat type were the greatest predictors of similarity among samples (adonis, lower clade *R*^2^= 0.228, *p* = 0.001, habitat type *R*^2^= 0.196, *p* = 0.001). Higher clade group explained much less variation (adonis, *R*^2^= 0.048, *p* = 0.001) (**Figure 5**). Weighted UniFrac analysis of all *Ensatina* samples yielded similar results as unweighted results, but with habitat type explaining slightly more variation than lower clade group (adonis, lower clade *R*^2^= 0.205, *p* = 0.001, habitat type *R*^2^= 0.221, *p* = 0.001, higher clade *R*^2^= 0.04, *p* = 0.001; **Figure 5**). Therefore, when only examining bacterial species present, lower clade group identity appears to have a larger influence on the microbiome composition than habitat type. However, when we account for the relative abundances of those bacterial species among samples, habitat type appears to play a larger role than lower clade group identity. We also tested for a correlation between genetic distance and Unifrac distance for a subset of lower clade groups for which genetic distances have been established (Wake, 1997; Kuchta et al., 2009b). We found that there was no significant correlation between genetic distance and unweighted Unifrac distance, nor weighted Unifrac distance (all *p*-values > 0.05).

We also did pairwise comparisons of unweighted and weighted Unifrac distances of *Ensatina* lower clade groups (**Table 2** and Supplementary Table 3). After Bonferroni correction, three lower clade comparisons showed significant differences in both weighted and unweighted Unifrac distances. Two comparisons were significantly different for weighted, but not unweighted, Unifrac distances. Ten other lower clade comparisons were significantly different for unweighted Unifrac distances but no longer significantly different when looking at weighted data. There was no pattern, in terms of clades with higher genetic distance being more likely to show distinct microbiomes.

**Table 2:** Summary of *R*^2^ and *p*-values of adonis analyses from *Ensatina* lower clade group pairwise comparisons of weighted and unweighted Unifrac distances.

Lower clade 1	Lower clade 2	Unweighted *R*^2^	Unweighted *p*-value	Weighted *R*^2^	Weighted *p*-value
Eschscholtzii	Klauberi	0.15755	0.001*	0.19898	0.001*
Eschscholtzii	Oregonensis [2]	0.10078	0.001*	–	0.172
Northern Platensis	Oregonensis [2]	0.1683	0.001*	0.23664	0.001*
Northern Platensis	Oregonensis [1]	0.13087	0.009	0.16953	0.001*
Northern Platensis	Eschscholtzii	0.15971	0.001*	0.23874	0.001*
Oregonensis [1]	Klauberi	0.1706	0.001*	0.16023	0.01
Oregonensis [2]	Klauberi	0.18177	0.001*	0.17119	0.005
Oregonensis [4]	Klauberi	0.2117	0.001*	–	0.165
Picta	Eschscholtzii	0.10022	0.002	0.21106	0.001*
Picta	Klauberi	0.11739	0.001*	0.14155	0.014
Xanthoptica [1]	Oregonensis [2]	0.17621	0.001*	0.1362	0.012
Xanthoptica [1]	Oregonensis [3]	0.14161	0.001*	–	0.112
Xanthoptica [1]	Eschscholtzii	0.12407	0.001*	0.12109	0.021
Xanthoptica [1]	Oregonensis [1]	0.14648	0.001*	0.09914	0.05
Xanthoptica [1]	Klauberi	0.11135	0.001*	–	0.219

*After Bonferroni correction, only p-values ≤ 0.001 (^∗^) were considered significant. Only significant comparisons are included in this table. For full results from all comparisons, see Supplementary Table 3.*

We determined that 29 OTUs made up the core microbiome of *Ensatina* (Supplementary Table 5). These OTUs were present in a minimum of 90% of *Ensatina* samples and made up 1.6–87.3% of the total microbiome for any given individual (mean = 38.6%). The average core abundance was non-normally distributed (Shapiro–Wilk test, *p* < 0.001) and varied between lower clade groups: *E. e. eschscholtzii* (53.9%), *E. e. klauberi* (26.4%), *E. e.* northern *platensis* (17.9%), *E. e. oregonensis* [1] (51.7%), *E. e. oregonensis* [2] (59.6%), *E. e. oregonensis* [3] (32.5%), *E. e. oregonensis* [4] (22.1%), *E. e. picta* (47.2%), *E. e.* southern *platensis* (26.6%), *E. e. xanthoptica* [1] (27.2%). Differences in core abundances were found to be significantly different across lower clade groups (Kruskal–Wallis test, *p* < 0.001).

### *Ensatina* vs. *Batrachoseps*

Measures of alpha diversity were not significantly different, with the exception of evenness, between samples collected from *Ensatina* and *Batrachoseps*, even after excluding *Ensatina* samples from geographic ranges that did not overlap with *Batrachoseps*. *Ensatina*’s alpha diversity was significantly more even between samples than *Batrachoseps* (non-parametric two-sample *t*-test, Shannon’s equitability, *p* = 0.045). When comparing all *Ensatina* samples to each species of *Batrachoseps* separately, *Ensatina* was significantly more even than *B. attenuatus* (non-parametric two-sample *t*-test, Shannon’s equitability, *p* = 0.03) and did not differ from the other two *Batrachoseps* species. However, when only considering *Ensatina* samples that overlapped with the range of *Batrachoseps* samples (**Figure 1** and **Table 1**), *Ensatina* was not significantly more even than *Batrachoseps*, nor any one particular species of *Batrachoseps*. Additionally, when comparing *Batrachoseps* and *Ensatina* within each site, we found no significant differences in any alpha diversity metrics. Comparing *Batrachoseps* species to one another, the only difference we found was that *B. gregarious* was slightly more even than *B. attenuatus* (non-parametric two-sample *t*-test, Shannon’s equitability, *p* = 0.036).

*Batrachoseps* had six dominant OTUs (≥3% of microbiome composition) and *Ensatina* had four, accounting for 40.5% and 26.3% of the overall microbiome community, respectively (**Figure 2**). *Batrachoseps* and *Ensatina* shared two dominant OTUs, both of which were from the family Pseudomonas. *Batrachoseps’* other dominant OTUs belonged to the family Xanthomonadaceae, and the genera *Sanguibacter, Achromobacter*, and *Chlamydia*. *Ensatina*’s other dominant OTUs were in the genera *Achromobacter* and *Chlamydia*. When comparing salamander genera there were 865 OTUs unique to *Ensatina*, making up 2.0% of the community, and there were 108 OTUs unique to *Batrachoseps*, making up 0.65% of the community. Looking at individual species within *Batrachoseps*, we found that *B. attenuatus* had seven dominant OTUs, *B. luciae* had five, and *B. gregarious* had two (**Figure 2**). These dominant OTUs made up 58.1%, 27.5%, and 14.2% of the microbiome communities for each of these three species, respectively.

When analyzing beta diversity among samples, genus explained a very small amount of variation between all *Ensatina* and *Batrachoseps* samples in both unweighted (adonis *R*^2^= 0.014, *p* = 0.017) and weighted analyses (adonis *R*^2^= 0.033, *p* = 0.001) (**Figure 3**). However, when we excluded *Ensatina* samples from outside the geographic range where we found *Batrachoseps*, genus explained no significant variation in samples using an unweighted analysis (adonis *p* > 0.05), and explained a small amount of variation using a weighted analysis (adonis *R*^2^= 0.027, *p* = 0.032). We also compared *Ensatina* and *Batrachoseps* samples within each site. After Bonferroni correction, to account for the six pairwise comparisons, genus did not explain within site variation between salamander samples (all *p*-values > cut-off of 0.008).

Of the 29 core OTUs identified in *Ensatina* (Supplementary Table 5), 16 of them belonged to the family Pseudomonadaceae. The other core OTUs belonged to the families: Comamonadaceae, Methylobacteriaceae, Sanguibacteraceae, Microbacteriaceae, Propionibacteriaceae, Bradyrhizobiaceae, Sphingomonadaceae, Xanthomonadaceae, Enterobacteriaceae, and Alcaligenaceae. We identified 95 OTUs found on 90% of the *Batrachoseps* individuals sampled (Supplementary Table 5). It is important to note that these 95 OTUs should not be considered a true representation of the core microbiome for this genus, as we only sampled three of the 21 species of *Batrachoseps*. Within *Batrachoseps attenuatus* we found 67 core OTUs. The core microbiome for this salamander species made up an average of 70.5% of the total microbiome, varying between 14.3% and 93.2% of the microbiome per individual.

## Discussion

In this study, we characterized the skin microbiome of the plethodontid salamander *Ensatina eschscholtzii* throughout its range. Our analysis included 10 of the 12 lower clade groups, as well as the three higher clade groups, that make up the *Ensatina* species complex (Kuchta et al., 2009a). Additionally, we characterized the microbiome across three species in the genus *Batrachoseps*, which belongs to the same family (Plethodontidae) as *Ensatina.* We compared microbiome variation between groups of salamanders of different genetic distances to evaluate the potential for the skin microbiome to serve as a phylogenetic signal in these terrestrial salamanders. We also evaluated microbes present in the soil and various habitat variables to elucidate the respective roles of host and their surrounding environment in shaping the skin microbiome community.

### Differences Between Soil and Salamanders

Soil had significantly higher alpha diversity than salamander samples. This is unsurprising given that the amount of microbes associated with organisms is predicted to be much less than the alpha diversity found within soil (Whitman et al., 1998; Curtis et al., 2002). It is important to note that while we did find a significant difference in alpha diversity, the magnitude of this difference was not as great as one might expect. The alpha diversity we observed in our soil samples is likely an underrepresentation of the full microbial diversity present in our terrestrial salamanders’ environment. For each *Ensatina* lower clade group, we only analyzed up to 5 soil samples, versus up to 15 salamander samples (10 *Ensatina* and 5 *Batrachoseps*). Additionally soil samples were collected from the very top surface level, rather than taking a deeper core sample, which would show greater diversity. This top layer of collected substrate was often predominated with decaying wood matter rather than silt, clay. or sand. Previous evaluation of bacterial communities on terrestrial salamanders versus free-living assemblages found on cover objects (i.e., decaying logs) identified similar magnitudes of difference in alpha diversity as reported here, with logs actually having lower richness than salamander skin (Fitzpatrick and Allison, 2014). Our soil samples were also localized from under cover objects, which may explain why we found many more unique OTUs on salamanders than within the soil, especially considering salamanders move through their environment, exposing them to a broad spectrum of substrates and surfaces.

We also found that soil samples exhibited more evenness in alpha diversity than salamander samples. High species diversity and uniform species distribution in soil can be explained by low competition between bacterial species due to spatial heterogeneity of resources (Zhou et al., 2002). The high diversity we observed in soil explains, in part, why we found no dominant OTUs (≥3% relative abundance) within soil samples. Other studies have also found high alpha diversity to be associated with high evenness across bacterial species in the soil (Rubin et al., 2013).

It is notable that salamanders and soil shared no dominant OTUs from the same genera. Two dominant salamander OTUs belonged to the genus *Pseudomonas*. Many species in this genus have been identified as amphibian symbionts that protect against disease, including chytridiomycosis (Harris et al., 2006; Flechas et al., 2012; Woodhams et al., 2015), which may explain why none of the salamanders sampled in this study were positive for *Batrachochytrium dendrobatidis*. Several studies corroborate a lower susceptibility of salamanders to *Batrachochytrium dendrobatidis*, which could be explained in part by their symbiotic bacteria (Bancroft et al., 2011; Muletz et al., 2014; Sette et al., 2015). Alternatively, the salamanders evaluated in this study may also exhibit lower levels of susceptibility to *Batrachochytrium dendrobatidis* due to their terrestrial life history limiting their contact with this pathogen, or due to environmental conditions (i.e., drought) during this study, which was likely to inhibit fungal growth. It is unclear what potential symbiotic function dominant OTUs in the genera *Achromobacter* and *Chlamydia* found on *Ensatina* skin might play; however, species within *Chlamydia* have been identified as pathogenic to salamanders (Martel et al., 2012).

In our analyses of beta diversity using the unweighted UniFrac metric, sample type (soil or salamander) was not the strongest predictor of microbiome composition across all samples. Instead, site and habitat type explained the most variation. This indicates that the geographic location in which these salamanders live is playing a role in the microbes present in the environment and therefore affecting which microbes are available for acquisition by the host salamanders. However, interestingly, when relative abundances of the bacterial species present were considered (weighted UniFrac), sample type (soil or salamander) was the strongest predictor of microbiome composition. These results suggest that salamanders are likely procuring many bacterial species from their surroundings, but that salamander skin provides different conditions than the soil, allowing for skin and soil microbial assemblages to vary in relative species abundances, supporting previous findings (Fitzpatrick and Allison, 2014; Walke et al., 2014; Prado-Irwin et al., 2017). The skin’s conditions for bacterial growth are likely different from the conditions provided in the environment. Indeed, soil microbial community richness and diversity have been strongly driven by pH conditions (Fierer and Jackson, 2006), whereas microbial communities on amphibian skin are influenced more by other factors such as diet (Antwis et al., 2014), immune defenses, and temperature (Woodhams et al., 2014).

### Influences of Host Identity on the Cutaneous Microbiome

After establishing that salamander cutaneous microbial community composition is distinct from the surrounding soil, we sought to explore the extent to which host identity might be able to explain variation in the microbiome within salamanders. We first evaluated differences between the *Ensatina* lower clade groups. Overall, there was a general lack of differences in alpha diversity of the skin microbiome between the *Ensatina* clade groups. A previous study comparing subspecies on an aquatic salamander, *Cryptobranchus alleganiensis*, found a similar lack of differentiation in alpha diversity among conspecific hosts (Hernández-Gómez et al., 2017).

Lower clade group identity did indicate differences in the beta diversity of the microbial community. However, most pairwise comparisons of *Ensatina* lower clades showed no differences in unweighted or weighted analyses, and only three comparisons were significantly different for both metrics. Therefore, the role of lower clade group identity in explaining differences in beta diversity was likely driven by a few unique microbiomes of particular clades, rather than each clade being unique from all others (**Table 2**). Genetic distances between *Ensatina* subspecies have been previously described (Wake and Yanev, 1986; Kuchta et al., 2009b) and our comparisons of beta diversity did not show any pattern related to genetic distance between lower clade groups. For example, *E. e. eschscholtzii* and *E. e*. southern *platensis* are more distantly related than *E. e.* northern *platensis* and *E. e. oregonensis* [2], but only the latter comparison showed significant differences in beta diversity. Despite the fact that we did not see a correlation between differentiation in the microbiome and genetic distance of hosts, it is interesting to note that the two most distantly related clade groups, *E. e. klauberi* and *E. e. eschscholtzii*, did show significant differences in both the weighted and unweighted analyses of beta diversity. However, a previous study comparing populations within a single subspecies (*E. e. xanthoptica*) did not find a correlation between genetic distance and beta diversity (Prado-Irwin et al., 2017).

Habitat type also predicted the microbial community. Due to the fact that the clade identity is correlated with habitat type, it is possible that the differences we observed in the microbial communities between lower clade groups are driven by habitat differences rather than traits (or evolutionary history) of the salamanders themselves. Further support for this conclusion comes from the analysis of higher-order clades (Coastal Clade, Clade A, Oregonensis Clade), which span larger geographic ranges and more habitat types. In our analysis of beta diversity at the higher clade level, host identity does not explain a large amount of variation between samples (**Figure 5**), lending credence to the conclusion that microbiome differences between these higher-order clades are likely more driven by habitat type. Previous studies have found that environmental variables such as land use type and elevation are important predictors of amphibian skin microbiome beta diversity (Hughey et al., 2017). Additionally, an experiment on salamander larvae showed that the skin microbiome shifts in response to transplantation into a different aquatic habitat (Bletz et al., 2016).

Due to the fact that different *Ensatina* clades are necessarily correlated to localities, we included *Batrachoseps* in our study, which are sympatric with *Ensatina* for much of their range, as a method of control. If the microbiomes of *Ensatina* clade groups were distinct, and *Ensatina* and *Batrachoseps* microbiomes were even more differentiated, this would provide strong evidence that host identity was contributing to differences in microbiome composition. However, as mentioned prior, we did not find *Ensatina* clade group (higher or lower) to be a strong predictor of microbiome variation. Instead, we were able to use our *Batrachoseps* data to evaluate if species identity within a family could serve as a predictor of microbiome composition for samples paired within a site. Studies on aquatic amphibians of different families have found species identity to be a significant predictor of the microbiome composition (McKenzie et al., 2012; Kueneman et al., 2014). We sought to determine if a similar pattern could be observed in California terrestrial salamanders within a family. This would help us determine which taxonomic level is most relevant for evaluating differences in the microbiome.

We found no differences in alpha diversity between *Ensatina* and *Batrachoseps*, and no differences in beta diversity when comparing sympatric *Ensatina* and *Batrachoseps* samples (**Figure 3**). From this, we conclude that taxonomic identity within these groups is not a reliable predictor of the microbial community and that any differences between these hosts due to size, diet, habitat use, etc. do not strongly influence the microbial communities that can reside on the skin. These results differ from what has been found in aquatic amphibians, where host species has been shown to be the strongest predictor of differences in skin microbiome samples, as opposed to site effects (McKenzie et al., 2012; Kueneman et al., 2014). This disparity may be explained by differences in aquatic and terrestrial amphibians, with major differences in host ecology driving patterns of microbiome composition and influencing the relative role of host phylogenetic effects (Bletz et al., 2017). Alternatively, it may be that *Batrachoseps* and *Ensatina* are not genetically differentiated enough to detect host effects, as found in aquatic amphibian communities. However, other work on plethodontids corroborates what we found (Muletz Wolz et al., 2017), while work on subspecies of aquatic salamanders show differences in beta diversity (Hernández-Gómez et al., 2017). We might expect plethodontids in particular to experience similar selective pressures on their skin microbiomes, and therefore exhibit more uniformity across species, due to the fact that they are lung-less and therefore rely heavily on their skin for biological processes.

Though we did not find that host phylogeny strongly influences microbiome variation in our system, more studies must be done to determine if this is true across other systems, including within amphibians at higher taxonomic levels. Studies done on primates have found evidence that differences in the gut and skin microbiome coincide with divergences between host species (Council et al., 2016; Moeller et al., 2016). A recent study on aquatic amphibians found that the skin microbiome beta diversity differed between host orders but not host species within the same order (Bletz et al., 2017). However, it can be difficult to determine if differences are due to host genetics or other factors such as diet (Ley et al., 2008b). Though studies have shown species identity to be connected to microbiome composition in amphibians, it is unclear which host factors drive those differences. Future studies should try and explore the respective roles of genetics, environment and the interaction between the two in shaping the microbiome.

While we did not find strong evidence that the microbial communities are significantly different between any taxonomic groups, we did identify unique OTUs to each genus, species, and clade group. Though collectively the unique OTUs only accounted for small proportions of the total microbiome, they could be playing an important role in host health (Podar et al., 2007; Hajishengallis et al., 2011; Human Microbiome Project Consortium, 2012; Davenport et al., 2014). The functionality of rare versus dominant antifungal symbionts differs depending on the host species (Walke et al., 2017). However, other studies have found that low abundance microorganisms do not play any important function in host physiology, and therefore the unique OTUs we found may be insignificant (Lee et al., 2016).

## Conclusion

The primary aim of this study was to determine whether differences in amphibian skin microbial communities could be explained due to host divergence leading to deviating microbial acquisition. If this occurs, we would expect more distantly related hosts to have more distinct microbiomes, and more closely related hosts to have more similar microbiomes. Our study did not support this hypothesis for two plethodontid genera. Differences in beta diversity were low between genera and between intra-generic *Ensatina* clades, with taxonomic group explaining little to no variation among samples. While we did find that lower clade group was a predictor of microbiome composition, habitat type explained a similar amount of variation. Our results suggest that aspects of the environment that contribute to habitat type influence the skin microbiome of plethodontids, and host specific factors may also be playing a role. Further studies need to be done to fully detangle the relative impacts of the host vs. the environment and explore how specific environmental factors influence the skin microbiome.

## Author Contributions

AB, AZ, and VV conceived and designed the study. AB and SP-I collected data and performed the lab work. AB analyzed the data and primarily wrote the manuscript with input regarding interpretation and drafts from AZ, VV, and SP-I.

## Conflict of Interest Statement

The authors declare that the research was conducted in the absence of any commercial or financial relationships that could be construed as a potential conflict of interest.

## Footnotes

1 www.databasin.org

2 http://greengenes.lbl.gov
